# Omission of nursing care in hospitalization units*

**DOI:** 10.1590/1518-8345.3138.3233

**Published:** 2020-02-14

**Authors:** Juliana Carvalho de Lima, Ana Elisa Bauer de Camargo Silva, Maria Helena Larcher Caliri

**Affiliations:** 1 Universidade Federal de Goiás, Faculdade de Enfermagem, Goiânia, GO, Brazil.; 2 Universidade de São Paulo, Escola de Enfermagem de Ribeirão Preto, PAHO/WHO Collaborating Centre for Nursing Research Development, Ribeirão Preto, SP, Brazil.

**Keywords:** Nursing, Nursing Care, Patient Safety, Quality of Health Care, Risk Management, Inpatient Care Units, Enfermagem, Cuidado de Enfermagem, Segurança do Paciente, Qualidade da Assistência à Saúde, Gestão de Risco, Unidades de Internação, Enfermería, Atención de Enfermería, Seguridad del Paciente, Calidad de la Atención de Salud, Gestión de Riesgos, Unidades de Internación

## Abstract

**Objective:**

to describe the prevalence and reasons for omission of nursing care, according to the perception of nursing professionals working in a teaching hospital.

**Method:**

a cross-sectional study was carried out with 267 professionals from ten hospitalization units. Data were collected by the MISSCARE-Brasil instrument. Descriptive statistics and Pearson’s Chi-square or Fisher’s exact tests were used to compare differences in the prevalence of omission among professional categories.

**Results:**

among the elements of nursing care, the highest prevalence of omission consisted in: *to sit up the patient out of bed* (70.3%), *ambulation three times a day* (69.1%), and *participation in the discussion of the interdisciplinary team on patient’s health care* (67.2%). The most frequent reasons were: inadequate number of staff (85.4%), inadequate number of staff for providing care or in administrative tasks (81.6%), and unexpected increase in the number and/or greater severity of patients (79.8%). Nurses reported major omission than nursing technicians/auxiliaries in four elements of care (p<0.05).

**Conclusion:**

according to our study, there is high prevalence of omission of nursing care elements from the professionals’ perspective. Factors related to human and material resources were more reported as causes for such omission.

## Abstract (pt)

**Objetivo:**

descrever a prevalência e as razões da omissão de cuidados de enfermagem, segundo a percepção de profissionais de enfermagem de um hospital de ensino.

**Método:**

estudo transversal realizado com 267 profissionais de dez unidades de internação. Os dados foram coletados por meio do instrumento MISSCARE-Brasil. Utilizou-se estatística descritiva e testes qui-quadrado de Pearson ou exato de Fisher para comparar diferenças na prevalência de omissão entre as categorias profissionais.

**Resultados:**

dentre os elementos do cuidado de enfermagem, as maiores prevalências de omissão foram: *sentar o paciente fora do leito* (70,3%), *deambulação três vezes ao dia* (69,1%) e *participação em discussão da equipe interdisciplinar sobre a assistência ao paciente* (67,2%). As razões mais frequentes foram: número inadequado de pessoal (85,4%), número inadequado de pessoal para a assistência ou tarefas administrativas (81,6%) e aumento inesperado no volume e/ou gravidade dos pacientes (79,8%). Os enfermeiros relataram maior omissão do que os técnicos/auxiliares de enfermagem em quatro elementos do cuidado (p<0,05).

**Conclusão:**

o estudo evidencia alta prevalência de omissão de elementos do cuidado de enfermagem na perspectiva dos profissionais. Fatores relacionados aos recursos humanos e materiais foram mais relatados como causas para esta omissão.

## Abstract (es)

**Objetivo:**

describir la prevalencia y las razones de la omisión de cuidados de enfermería, según la percepción del personal de enfermería de un hospital de enseñanza.

**Método:**

estudio transversal realizado con 267 profesionales de diez unidades de internación. Los datos se recopilaron por medio del instrumento MISSCARE-Brasil. Se emplearon estadística descriptiva y prueba chi cuadrado de Pearson o exacto de Fisher para comparar diferencias en la prevalencia de omisión entre las categorías profesionales.

**Resultados:**

entre los elementos del cuidado de enfermería, las mayores prevalencias de omisión fueron: *sentar al paciente fuera de la cama* (70,3 %); *deambulación tres veces al día* (69,1 %) y *participación en la discusión del equipo interdisciplinar sobre la asistencia al paciente* (67,2 %). Las razones más frecuentes fueron: el número inadecuado de personal (85,4 %); el número inadecuado de personal para la asistencia o tareas administrativas (81,6 %) y el aumento inesperado del volumen o gravedad de los pacientes (79,8 %). Los enfermeros relataron una mayor omisión que los técnicos/auxiliares de enfermería en cuatro elementos del cuidado (p<0,05).

**Conclusión:**

el estudio pone de manifiesto la alta prevalencia de omisión de elementos del cuidado de enfermería desde la perspectiva de los profesionales. Factores relacionados con los recursos humanos y materiales han sido los más relatados como causas para esta omisión.

## Introduction

Ensuring the quality of the care provided to patients and their safety in healthcare institutions has been a challenge considering evidence of errors when providing this care. Such errors can be manifested in two ways: errors of commission, when the planned action is wrongly performed; and errors of omission, when the right action cannot be performed^(^ 1 ^)^.

The omission of nursing care refers to the lack or delay in providing any care-related aspect demanded by the patient (partly or completely)^(^ 2 ^)^. Such error has been presented as a common, universal, and frequent issue due to systemic factors^(^ 3 ^)^.

Specifically in nursing care, the omission phenomenon can be explained by the “Missed nursing care” model, according to which the structure of organizations, such as characteristics of the hospital, unit, and staff, interferes in the work process of nursing, resulting in omitted care and in negative consequences for nursing professionals, such as dissatisfaction and absenteeism, and for patients such as infections, falls, pressure injury, among others^(^ 4 ^-^ 5 ^)^.

Considering studies conducted in England, Mexico, and the United States of America (USA), the most omitted care by nursing professionals consist in: ambulation; oral hygiene; talking and comforting patients; participating in interdisciplinary discussions; healthcare planning; and education of patients and relatives^(^ 3 ^,^ 6 ^-^ 10 ^)^.

In Brazil, authors of studies have evidenced that the care directed to meeting emotional, spiritual, and social needs, bowel movement, and physical safety of the adult patient were omitted and/or poorly performed during hospitalization^(^ 11 ^)^. A similar datum was verified in a pediatric context, regarding the provision of orientations to the companions of hospitalized children and the follow-up of the children’s shower^(^ 12 ^)^.

The omission of care brings undesirable consequences to patients, professionals, and healthcare institutions. Negative results for patients have been associated with the omission of nursing care, such as: pressure injury, medication errors, falls, infections^(^ 4 ^,^ 13 ^)^, readmissions^(^ 9 ^)^, and even death^(^ 14 ^)^.

Regarding nursing professionals, the awareness of not having managed to provide their patients with all the necessary care may generate dissatisfaction, increased intention to quit the job, and the Burnout Syndrome^(^ 5 ^)^. In their turn, healthcare institutions have their cost increased due to the increase in the length of stay of patients, readmissions, and the need for repair/treatment of damages caused to the patients^(^ 5 ^)^.

Evidence of nursing care omission justify the need to understand its reasons. Aspects related to human resources, material resources, and communication have been the most prevalent factors that make professionals unable to perform all the care required by the patients^(^ 15 ^)^.

It is believed that the identification, mitigation, and transparent discussion on omissions of nursing care can assist in the management of institutional risk and in the creation of a safety culture, consisting in an “early warning” of greater risk to negative results of patients^(^ 14 ^)^.

Authors of studies on the omission of nursing care may indicate paths and solutions for preventing this type of care failure and assisting in the planning of corrective actions, impacting the improvement of care quality and safety. Understanding the phenomenon of omission of care is also important for teaching in nursing, making professors and students aware of this occurrence and of the development of prevention strategies.

Therefore, we raised the following research question: which nursing care is omitted the most by nursing professionals and what are the most prevalent reasons for this omission? Hence, we aimed to describe the prevalence and reasons for omission of nursing care, according to the perception of nursing professionals working in a teaching hospital.

## Method

This is a cross-sectional study carried out in ten hospitalization units of a public teaching hospital in Goiás, namely: Medicine Unit, Surgical Unit, Pediatric Unit, Maternal Infant Unit, Orthopedic Unit, Tropical Unit, Intensive Care Unit (ICU), Surgical ICU, and Neonatal ICU.

The study population consisted of all nurses, nursing technicians and auxiliaries who have been working for over one month in the investigated units during the data collection period. Professionals in management positions responsible for more than one unit were excluded, in addition to those who were on leave during the data collection period and those who did not perform nursing actions.

During the data collection period, the institution had 626 nursing professionals and 401 of them worked in the selected hospitalization units. Of these, 376 met the inclusion criteria, 47 refused to participate in the research, and 62 did not return the data collection instruments.

Data were collected from April 15 to December 23, 2017, by the MISSCARE-Brasil instrument, a translated and validated instrument for the Brazilian culture^(^ 16 ^)^. The instrument was used with authorization and guidance provided by the author.

MISSCARE-Brasil is a self-applicable questionnaire, consisting of three parts. The first part gathers questions about general information about the participants and the workplace. The second part of the instrument, or part A, consists of 28 items regarding the elements of omitted nursing care practices, with Likert-type responses, varying from “it is never performed” to “it is always performed.” And the third part of the instrument, or part B, gathers 28 items concerning the reasons for not performing nursing care, with Likert-type responses varying from “significant reason” to “is not a reason for the omission of care.”

Participants were approached and instructed to respond the instrument outside the location and not during working hours and to return it later, according to the date preestablished by the researcher. All of them were instructed on how to respond to the questionnaire, guaranteeing their anonymity and respecting the refusals.

Data were double-checked and typed in a spreadsheet, and analysis was performed using the *Statistical Package for Social Science* (SPSS) software version 24.0. Prior to the analysis, we transposed the response codes regarding the items of parts A and B; thus, the higher values corresponded to higher levels of omission and the most important reasons^(^ 16 ^)^. 

After reversal, responses were dichotomized and, hence, the alternatives “it is occasionally omitted,” “it is rarely performed,” and “it is never performed” mean omitted care, and the alternatives “it is often performed” and “it is always performed” represent the performed care. Answers regarding the reasons were also dichotomized, considering as reason for omission the options “significant reason” and “moderate reason,” and as no reason for omission, “little significant reason” and “not a reason”^(^ 6 ^,^ 16 ^-^ 17 ^)^.

Statistical analysis calculations were performed with the aid of SPSS software version 24.0. Descriptive analysis of the quantitative variables consisted in mean, standard deviation (SD), median, interquartile range (IQR), minimum, and maximum. The qualitative variables (educational level, higher education level, position, work period, shift in which more frequently works) were presented as absolute and relative frequencies.

The prevalence of omission of each provided care was calculated by dividing the number of omitted care practices by the total amount of responses that such nursing care element obtained, multiplied by 100. It is noteworthy that the answer “does not apply” was not included in the prevalence.

Similarly, the prevalence of reasons for omission of care was calculated by dividing the number of responses considered as a reason for omission by the total amount of responses that such reason obtained, multiplied by 100.

To verify differences in the prevalence of omission of nursing care per professional category (nursing technicians and auxiliaries × nurses), we used Pearson’s Chi-squared or Fisher’s exact tests.

The internal reliability of MISSCARE-Brasil was evaluated by the standardized Cronbach’s alpha, and values above 0.7 were deemed acceptable^(^ 18 ^)^.

This research was approved by the Research Ethics Committee of the institution, via Plataforma Brasil (Opinion no. 1,922,667), and followed the recommendations proposed by the National Health Council, in Resolution 466/2012^(^ 19 ^)^, which regulates research involving human beings. All professionals who agreed to participate in the study received the Informed Consent Form and were asked to read and sign it.

## Results

A total of 267 nursing professionals participated in the study, corresponding to a response rate of 71.0% of the population, and 87.6% were women, with a median age of 43.0 years (IQR: 15), mean of 43.1 (SD: 10.1), and varying from 23 to 70 years.

The analysis of the professional category and position occupied in the unit pointed out that 177 (66.3%) were nursing technicians, 11 (4.1%) were nursing auxiliaries, 72 (27.0%) were nurses, and 7 (2.6%) were nurses occupying administrative positions. Hence, there were 79 (29.6%) nurses and 188 (70.4%) nursing technicians and auxiliaries.

In relation to education level, 77.5% of the professionals hold an undergraduate and/or graduate degree. Daytime working period (55.8%) and 12-hour shifts (78.3%) were the most prevalent.

The analysis of the reliability of responses of the MISSCARE-Brasil instrument, performed by Cronbach’s alpha coefficient (α =), indicated acceptable values, with internal consistency α = 0.913 for part A of the instrument, referring to the elements of nursing care, and α = 0.941 in part B, consisting in the reasons for the omission of care.

The nursing care practices with major prevalence of omission were *to sit up the patient out of bed* (70.3%), *ambulation three times a day* (69.1%), *participation in the discussion of the interdisciplinary team on patient’s health care* (67.2%), and *planning and teaching of the patient and/or family for hospital discharge* (51.1%) (Table 1).

**Table 1:** Prevalence of omission of nursing care. Goiânia, GO, Brazil, 2017

Omitted nursing care elements	n (%)
To sit up the patient out of bed	180 (70.3)
Ambulation three times a day or as prescribed	172 (69.1)
Participation in discussions of the interdisciplinary team on patient's health care	178 (67.2)
Planning and teaching of the patient and/or family for hospital discharge	136 (51.1)
Emotional support provided for the patient and/or family	93 (35.1)
Changing the patient's lying position every 2 hours	79 (29.7)
Complete record of all required data in the patient's medical records	77 (28.8)
Oral hygiene	74 (27.8)
Evaluation of the effectiveness of the administered medications	71 (26.6)
Fluid balance control	71 (26.7)
Providing meals to patients who feed by themselves	70 (28.0)
Responding to patient's call within 5 min	70 (27.7)
Orientations to patients and relatives regarding routines, procedures, and provided care	69 (25.8)
Administration of medications within 30 min prior to or after the prescribed time	65 (24.3)
Focused revaluation, according to the patient's condition	64 (24.0)
Evaluation of patient's conditions at each shift, identifying their care needs	53 (19.9)
Promptly sanitizing the patient after each bowel movement	50 (18.9)
Requests for administration of medications prescribed as "if necessary (Y/N)" are complied with in 15 min	43 (16.5)
Use of preventive measures for patients at risk of falling	38 (14.5)
Care provided by venous access and infusion, according to the norms of the institution	31 (11.6)
Hydrate the patient by providing oral fluids or by administering the probe tube	29 (11.1)
Evaluation of vital signs as prescribed	28 (10.5)
Airway aspiration	24 (12.7)
Feeding the patient or administering the diet by probe tube, at the proper time	24 (9.0)
Care provided to skin injuries/wounds	21 (7.9)
Patient's bath/hygiene/measures for preventing skin injuries	16 (6.0)
Sanitizing your hands	16 (6.0)
Monitoring capillary blood glucose	7 (2.6)

The omission of nursing care varied according to professional category in five elements of care (p< 0.05), considering that four of them were the most mentioned on the part of nurses, and *care provided to skin injuries/wounds* was the most mentioned on the part of nursing technicians and auxiliaries (Table 2). 

**Table 2:** Comparison of the omission of nursing care, according to nurses and nursing technicians and auxiliaries. Goiânia, GO, Brazil, 2017

Omitted nursing care practices	N* (N = 79)			TA^†^ (N = 188)		p-value^‡^
	Total^§^	n	%	n	%	
To sit up the patient out of bed	256	54	74.0	126	68.9	0.418
Participation in discussions of the interdisciplinary team on patient's health care	265	52	67.5	126	67.0	0.936
Ambulation three times a day	249	47	66.2	125	70.2	0.535
Planning and teaching of the patient and/or family for hospital discharge	266	37	46.8	99	52.9	0.363
Change the patient's lying position every 2 hours	266	34	43.6	45	23.9	0.001
Complete record of data in the patient's medical records	267	32	40.5	45	23.9	0.006
Responding to patient's call within 5 min	253	28	37.8	42	23.5	0.020
Emotional support provided for the patient and/or family	265	28	35.9	65	34.8	0.860
Oral hygiene	266	27	34.6	47	25.0	0.111
Evaluation of the effectiveness of medications	267	26	32.9	45	23.9	0.130
Fluid balance control	266	22	28.2	49	26.1	0.719
Providing meals to patients who feed by themselves	250	21	28.8	49	27.7	0.862
Orientations to patients and relatives regarding routines, procedures, and provided care	267	20	25.3	49	26.1	0.899
Administration of medications within 30 min prior to or after the prescribed time	267	18	22.8	47	25.0	0.700
Requests for administration of medications prescribed as "if necessary (Y/N)" are complied with in 15 min	261	17	21.8	26	14.2	0.130
Focused revaluation, according to the patient's condition	267	16	20.3	48	25.5	0.356
Promptly sanitizing the patient after each bowel movement	265	16	20.8	34	18.1	0.611
Evaluation of patient's conditions at each shift, identifying their care needs	267	15	19.0	38	20.2	0.819
Care provided with venous access and infusion	267	15	19.0	16	8.5	0.015
Use of preventive measures for patients at risk of falling	262	12	15.4	26	14.1	0.792
Evaluation of vital signs as prescribed	267	11	13.9	17	9.0	0.235
Airway aspiration	267	10	12.7	24	12.8	0.981
Feeding the patient or administering the diet by probe tube, at the proper time	266	10	12.8	14	7.4	0.164
Hydrate the patient by providing oral fluids or by administering the probe tube	261	9	11.8	20	10.8	0.810
Patient's bath/hygiene/measures for preventing skin injuries	266	5	6.4	11	5.9	1.000
Sanitizing your hands	267	5	6.3	11	5.9	1.000
Monitoring capillary blood glucose	267	3	3.8	4	2.1	0.426
Care provided to skin injuries/wounds	267	1	1.3	20	10.6	0.009

* N = Nurses;

† TA = Nursing technicians and auxiliaries;

‡ Pearson's Chi-square or Fisher's exact test;

§ To analyze the differences in the prevalence of omission per category, we excluded cases with the response "does not apply"

The inadequacy of human resources and the unexpected increase in the number and/or greater severity of patients were the most prevalent reasons for omission (Table 3).

**Table 3:** Prevalence of reasons for omission of nursing care. Goiânia, GO, Brazil, 2017

Reasons for omission of nursing care	n (%)
Inadequate number of staff	228 (85.4)
Inadequate number of staff for care-related or administrative tasks	218 (81.6)
Unexpected increase in number and/or greater severity of patients in the unit	213 (79.8)
Medications were unavailable when requested	204 (76.4)
Patient's emergency conditions	204 (76.4)
Materials/equipment did not properly work whenever necessary	203 (76.0)
Materials/equipment were unavailable whenever necessary	201 (75.3)
Other staff professionals did not provide care at the time when it was necessary	184 (68.9)
Many hospital admissions and discharges	173 (64.8)
Distribution of patients per professional is unbalanced	166 (62.2)
Tension/conflict or communication issues with other departments/support sectors	153 (57.3)
The transition from the previous shift or the units that refer patients is inadequate	153 (57.3)
The physical structure of the unit is inadequate, which makes it difficult to provide care to patients in isolation or in more distant areas	153 (57.3)
High number of professionals working were ill or with health problems	151 (56.6)
Tension/conflict or communication issues with the medical staff	144 (53.9)
Lack of education about the care to be provided	136 (50.9)
Lack of standardization to perform procedures/care practices	135 (50.6)
The nursing auxiliary did not report that the care has been provided	130 (48.7)
Professionals have more than one employment relationship, which reduces their commitment/attention/concentration to provide the care	127 (47.6)
Staff members do not help each other	125 (46.8)
Tension/conflict or communication issues with the nursing staff	114 (42.7)
The professional responsible for providing the care was outside the unit/sector or was unavailable	114 (42.7)
The professional who did not provided the care is not afraid of punishment/dismissal due to the stability in employment	108 (40.4)
The professional has no ethical posture and has no commitment and involvement with the work and/or with the institution	101 (37.8)
Lack of preparation on the part of nurses to lead, supervise, and conduct teamwork	100 (37.5)
The nursing professional is negligent (lazy, lacking attention or insensitivity)	98 (36.7)
Lack of motivation for work (due to low salary and/or lack of appreciation of the professional)	88 (33.0)
**Reasons for omission of nursing care**	**n (%)**
High number of nurses with little professional experience	77 (28.8)

## Discussion

Our results evidenced a high prevalence of omission of one or more nursing care practices. Such omission is an important variable to reflect on the need to revise the structure and work processes under development, which may be resulting in care-related outcomes lacking quality due to the non-provision of adequate treatment, in addition to causing damages to patients.

The internal consistency of the used instrument, the MISSCARE-Brasil, was considered acceptable, with results similar to other national^(^ 16 ^)^ and international^(^ 10 ^,^ 13 ^,^ 17 ^)^ studies, proving that it has a high reliability to measure the omission of nursing care.

We identified that *sitting up the patient out of bed* featured the highest prevalence (70.3%) of omission of care, followed by *ambulation of patients three times a day* (69.1%). The non-ambulation of the patients was also identified as the care with the highest prevalence of omission in studies conducted on nursing staff professionals in several countries^(^ 6 ^,^ 16 ^,^ 20 ^)^, and also by patients themselves (41.3%)^(^ 21 ^)^.

These two nursing care practices involve the mobilization of the patient out of bed. Mobilization of hospitalized patients provides physical benefits such as pain relief, decreased risk of deep vein thrombosis, decreased fatigue, prevention of recurrence of pneumonia and delirium, decreased risk of urinary tract infection, and improved physical functioning. In addition to social welfare, it improves quality of life, independence, it decreases anxiety, depressive mood, anguish, and increases patient comfort and satisfaction^(^ 22 ^)^. Organizational results can also be identified, such as the decrease in the length of stay, mortality of patients, and institutional costs^(^ 22 ^)^.

Evidences reflect the importance of mobilization for positive results in the reestablishment of patients and highlight the need for planning and developing methods and routines to ensure that this nursing action is systematically performed^(^ 22 ^)^.

Another care featuring high prevalence of omission (67.2%) was the *participation in the discussion of the interdisciplinary team on patient’s health care*. Multidisciplinary discussions provide better communication between the teams. The omission of this activity can interfere with the quality of care provided to the patient, because it does not allow exchange of information, collaboration, and reflections on therapeutic conducts to be performed, among the various professional categories that, daily, provide care to the same patient. The integrality of care, one of the doctrinary principles of the Brazilian Unified Health System, poses the challenge of implementing a form of work that enables interdisciplinarity, in such a way it effectively meets the several dimensions of the health-disease process and is able to address the complexity of the object of the health field^(^ 23 ^)^. However, in the current institutional culture of healthcare services, fragmentation, hierarchical relationships, individualized work per professionals, and resistance of a technical-scientific rationality prevail, as well as inequalities between specialties and the social valorization attributed to them^(^ 23 ^)^.

Nursing professionals, for not participating in discussions with professionals from other teams, miss opportunities to share the knowledge of those who accompany the patient 24 hours a day, and to highlight its importance for the patient’s recovery and provision of a quality care.

Moreover, we evidenced that another omitted care, with a prevalence of 51.1%, was the *planning and teaching of the patient and/or family for hospital discharge*. The failure in the education of patients and relatives is not only a Brazilian issue, since it was also evidenced in studies conducted in the USA^(^ 9 ^)^ and in Mexico^(^ 10 ^)^.

At a time when the active participation of patients and their relatives in their own care, to guarantee the patient’s safety^(^ 24 ^)^, is increasingly sought, patients’ lack of guidance and education in health is worrisome, and it may lead to complications in their health conditions and to readmission^(^ 4 ^)^. Teaching patients and relatives about their health condition and care to be provided after hospital discharge make patients to have greater adherence to treatment and feel engaged in their own care, preventing adverse events and avoiding unnecessary complications and readmissions^(^ 24 ^-^ 25 ^)^.

The comparison of the omission of nursing care, according to the perception of nurses and nursing technicians and auxiliaries, evidenced that there were differences between professional categories and, in some aspects of care, such differences were statistically significant. Overall, nurses perceived higher prevalence of omission of care than nursing technicians/auxiliaries concerning the *change in the patient’s lying position*, *record of data in the patient’s medical records*, *care provided with venous access and infusion*, and *responding to patient’s call within 5 min.* On the other hand, nursing technicians and auxiliaries perceived greater omission in the *care provided to skin injuries/wounds.* These differences must be better analyzed in further investigations.

Among the reasons for omission presented in the MISSCARE-Brasil instrument, the inadequacy of human resources and the unexpected increase in the number and/or greater severity of patients were perceived by the nursing professionals as of higher prevalence.

The inadequate number of staff and the unexpected increase in the number and/or greater severity of patients in the unit have been frequently mentioned in studies conducted by the nursing field whose authors seek to understand lack of quality, adverse events, and professional dissatisfaction^(^ 7 ^,^ 10 ^,^ 26 ^)^. The insufficient number of staff reflects in a greater number of patients per nursing professional and may impose burden to workers and generate higher rates of omitted care^(^ 27 ^)^.

Authors of a study in England identified that the number of patients per nurse was significantly associated with the omission of nursing care, and pointed out that when nurses provided care for a smaller number of patients, less care practices were omitted and the chance to omit some care practice also diminished^(^ 7 ^)^.

The National Institute for Clinical Excellence (NICE) proposed that the omission of care could be used as a “warning flag” at inadequate levels of staff and as an indicator of the quality of nursing services. It also reinforces the need for more evidence and indicators to determine safe levels of the nursing staff, in addition to studies in order to understand the extent to which they are actually achieved^(^ 28 ^)^.

Nursing professionals also perceived the lack of materials/equipment or inadequate functioning whenever necessary and the lack of medications as reasons or frequent causes for the omission of care. The management of material resources and the administration of these resources or supplies constitute the totality of material flows of a healthcare organization, consisting in a process with the following main activities: scheduling, purchasing, receiving, storage, distribution, and control^(^ 29 ^)^.

On the other hand, nurses are responsible for the management of material resources in hospitalization clinics, and by foreseeing, providing, being attentive to the quality of the material to be used, as well as the necessary amount, and monitoring its consumption, they can guarantee the quality of the provided care and avoid discontinuity of care. However, professionals are aware of issues related to financial resources in Brazilian public hospitals, which directly impact the acquisition of goods and lead to shortage of drugs, materials, and even equipment^(^ 30 ^)^.

Finally, the omission of nursing care practices evidenced in this study demonstrates a risk to the quality and safety of nursing care. The staff is often exposed to factors that prevent them from completing all necessary care for the patient, leaving them vulnerable. The work process of nursing professionals must be revised and redesigned, aiming at optimizing and defining the role, responsibilities, and workload of professionals in such a way that there is a work environment favorable to the continuity of care^(^ 31 ^)^.

Considering all these evidences, we understand that strategies to mitigate and prevent the omission of care must be implemented, because users of the healthcare system, called “patients” in many hospital organizations, need that all care practices for their treatment and recovery to be performed. However, omission of nursing care has been a silent phenomenon not perceived by the management. The first step is to acknowledge and discuss it, in order to understand its proportion. There must be leadership involvement along with the frontline professionals for planning improvement actions.

The main limitations of our study concern data collection performed in a single institution and the seasonal variation of the data collection period, which took place in eight months. However, it is worth noting that this is one of the first studies to perform a situational diagnosis of the omission of nursing care within the Brazilian context. It is noteworthy that this diagnosis is essential to awaken a differentiated and critical look at this frequent problem, but about which little or nothing was discussed until now. We suggest to carry out studies whose authors seek to analyze and compare the omission of care in other institutions and investigate factors that may influence in the non-performance of nursing care.

The relevance to professional practice in identifying and understanding reasons that lead to the occurrence of nursing care omission is in recognizing aspects of the nursing work process that require attention and decision-making on the part of the management of institutions, in such a way the continuity of care is ensured and adverse events due to lack of care are reduced.

## Conclusion

With this study we evidenced that the omission of care is a real and frequent phenomenon. The nursing care practices with major prevalence of omission were *to sit up the patient out of bed*, *ambulation three times a day*, *participation in the discussion of the interdisciplinary team on patient’s health care*, and *planning and teaching of the patient and/or family for hospital discharge*.

The most frequent reasons for the omission of nursing care were related to human resources and material resources. These reasons are focused on managerial and systemic failures, which should be analyzed and corrected in favor of the patient’s safety. Authors of studies, such as ours, evidence that nursing has been operating in situations unfavorable to the integral performance of the care process, demanding efforts to plan and adopt strategies to prevent the omission of care and improve care-related practices.
